# Distributions of Arctic and Northwest Atlantic killer whales inferred from oxygen isotopes

**DOI:** 10.1038/s41598-021-86272-5

**Published:** 2021-03-24

**Authors:** Cory J. D. Matthews, Fred J. Longstaffe, Jack W. Lawson, Steven H. Ferguson

**Affiliations:** 1 Fisheries and Oceans Canada, 501 University Crescent, Winnipeg, MB Canada; 2 Department of Earth Sciences, The University of Western Ontario, London, ON Canada; 3 Fisheries and Oceans Canada, 80 East White Hills Road, St John’s, NL Canada

**Keywords:** Ecology, Zoology

## Abstract

Killer whales (*Orcinus orca*) are distributed widely in all oceans, although they are most common in coastal waters of temperate and high-latitude regions. The species’ distribution has not been fully described in the northwest Atlantic (NWA), where killer whales move into seasonally ice-free waters of the eastern Canadian Arctic (ECA) and occur year-round off the coast of Newfoundland and Labrador farther south. We measured stable oxygen and carbon isotope ratios in dentine phosphate (δ^18^O_P_) and structural carbonate (δ^18^O_SC_, δ^13^C_SC_) of whole teeth and annual growth layers from killer whales that stranded in the ECA (n = 11) and NWA (n = 7). Source δ^18^O of marine water (δ^18^O_marine_) at location of origin was estimated from dentine δ^18^O_P_ values, and then compared with predicted isoscape values to assign individual distributions. Dentine δ^18^O_P_ values were also assessed against those of other known-origin North Atlantic odontocetes for spatial reference. Most ECA and NWA killer whales had mean δ^18^O_P_ and estimated δ^18^O_marine_ values consistent with ^18^O-depleted, high-latitude waters north of the Gulf Stream, above which a marked decrease in baseline δ^18^O values occurs. Several individuals, however, had relatively high values that reflected origins in ^18^O-enriched, low-latitude waters below this boundary. Within-tooth δ^18^O_SC_ ranges on the order of 1–2‰ indicated interannual variation in distribution. Different distributions inferred from oxygen isotopes suggest there is not a single killer whale population distributed across the northwest Atlantic, and corroborate dietary and morphological differences of purported ecotypes in the region.

## Introduction

Global distribution patterns of killer whales (*Orcinus orca*) indicate they are most abundant in coastal, high-latitude waters of both hemispheres, with lower densities occurring in less productive tropical and offshore waters^1^. In the northwest Atlantic (NWA), where there has been almost no directed research on killer whale distribution, incidental sightings and whaling records reflect a similar general pattern of decreasing abundance from the Arctic to the Gulf of Mexico and Caribbean Sea^2–4^. Over the past several decades, killer whales have been observed more frequently in both the eastern Canadian Arctic (ECA)^5^ and off the coast of Newfoundland and Labrador (NL)^6^, potentially reflecting shifting distributions and/or increasing abundance in response to declining sea ice^5^ or recovery from commercial whaling and culls^7^. Assessment of potential explanatory factors within the broader context of the NWA (e.g., where do these whales come from?), however, has been limited by existing observational data, which is largely qualitative and includes temporal and spatial biases.

Killer whales move into the seasonally ice-free waters of the ECA, where they are associated with summering aggregations of Arctic marine mammals^2,8,9^. They leave the region at the onset of sea ice formation for unknown overwintering areas^10,11^. Reeves and Mitchell^2^ suggested a possible continuous distribution between Canada and Greenland, but also hypothesized the range of ECA killer whales could extend along the North American coast as far south as the Caribbean, or into the open North Atlantic. Farther south, killer whales are sighted year-round off coastal NL, although less frequently during winter^6,12^. It is unclear whether this sightings pattern reflects seasonally variable observer effort, or a seasonal shift in distribution as has been documented in other killer whale populations^13^. Sightings of killer whales throughout the NWA during the summer months^4,14^, including both the ECA and NL^6,15^, provide evidence against a north–south migration, as does the lack of photo-identification matches between the two regions^16^. However, satellite telemetry^10^ and the presence of warm-water *Xenobalanus* barnacles^17^ indicate at least some killer whales observed in the ECA range into temperate and perhaps tropical waters.

One of the earliest ecological applications of stable isotope analysis has been the assignment of animal distributions through matching tissue isotope composition with underlying geographic isotope variation^18^. In both terrestrial and marine ecosystems, biogeochemical processes lead to systematic regional variation in baseline stable isotope composition known as ‘isoscapes’. For example, the interplay between physical and biological processes (e.g., air-sea gas exchange and primary productivity) affecting dissolved inorganic carbon (DIC) concentrations and associated ^13^C fractionation leads to pronounced latitudinal *δ*^13^C gradients in marine surface water carbon species and phytoplankton^19,20^. Baseline isotopic variation is incorporated into animal tissues via food and water (typically with some degree of isotopic discrimination that needs to be quantified and accounted for), thus serving as a marker of their geographic origins^18^. Although not commonly used in this context, the oxygen isotope composition (*δ*^18^O) of biogenic apatite is an effective marker of cetacean distributions across isotopically distinct regions of the ocean^21,22^. Marine surface water *δ*^18^O is governed by relative rates of evaporation and precipitation, resulting in a latitudinal gradient of several per mil in both hemispheres^19,23^. Cetacean body water closely tracks marine *δ*^18^O, as their dominant oxygen fluxes (transcutaneous water exchange, ingestion of water in food, and incidental or active ingestion of water while eating or drinking^24,25^) are not strongly fractionating^26^. Biogenic apatite in dentine and bone, in turn, precipitates in oxygen isotope equilibrium with a constant offset from body water in homeothermic mammals^21,27,28^, such that the *δ*^18^O of phosphate (*δ*^18^O_P_) and structural carbonate (*δ*^18^O_SC_) ultimately vary linearly with that of source marine water^21,22,29^.

The most pronounced variation in global marine *δ*^18^O occurs in the North Atlantic, where a latitudinal *δ*^18^O gradient spanning several per mil exists between the Arctic, which is characterized by ^18^O-depleted marine surface waters typical of high latitude regions, and the mid North Atlantic, which is characterized by ^18^O-enriched Gulf Stream waters^23^. The *δ*^13^C of DIC and phytoplankton also vary systematically across the North Atlantic, decreasing by up to 10 per mil moving northeast from the Caribbean to the Norwegian Sea^19,20^. These two elements together can thus provide complementary discriminatory power for evaluating North Atlantic whale distributions^30,31^. To that end, we measured *δ*^18^O_P_, *δ*^18^O_SC_, and *δ*^13^C of structural carbonate (*δ*^13^C_SC_) in biogenic apatite of dentine from killer whales that stranded in the ECA and NWA. Source marine water *δ*^18^O (*δ*^18^O_marine_) was estimated from dentine *δ*^18^O_P_ values using a published calibration^22^, and then compared to the North Atlantic *δ*^18^O isoscape^23^ to infer individual killer whale distributions. Our objectives were to evaluate whether ECA and NWA killer whale distributions (1) overlap within the broader North Atlantic, and (2) encompass the relatively ^18^O-enriched waters of the mid North Atlantic, as earlier hypothesised^2^ and recently demonstrated using satellite telemetry^10^. The *δ*^18^O values of most ECA and NWA killer whales indicated their distributions were restricted to the ^18^O-depleted, high-latitude waters surrounding the ECA, NL, and Greenland, although several individuals originated from a broader area encompassing ^18^O-enriched, low-latitude waters. These findings are discussed within the contexts of population structure and purported killer whale ecotypes in the ECA and NWA.

## Methods

### Specimen collection and sampling

**Figure 1:**
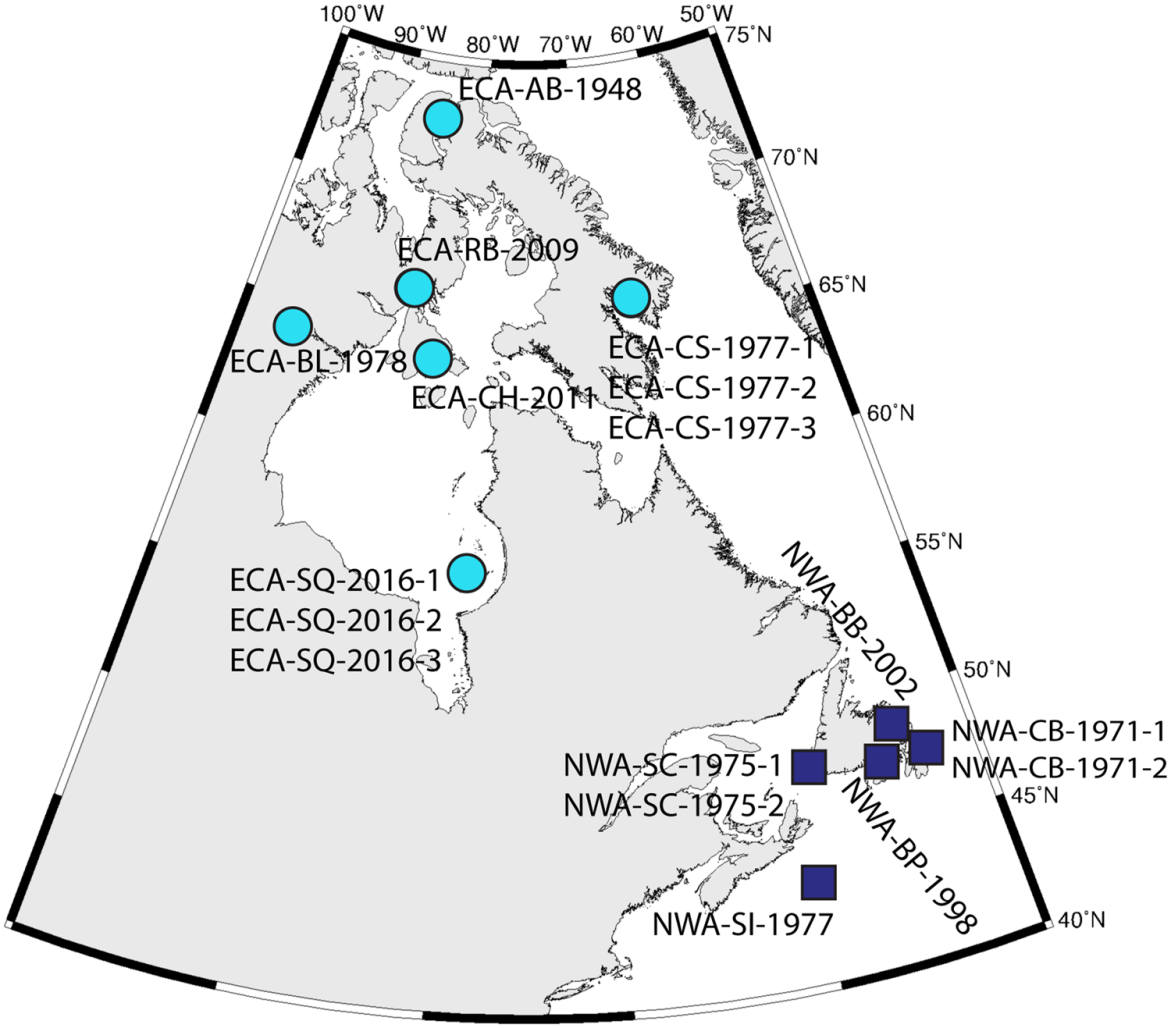
Locations of stranded killer whales in the eastern Canadian Arctic (ECA; turquoise circles) and Northwest Atlantic (NWA; purple squares) included in this study (specimen ID numbers match those presented in Tables 1 and 2). Map was created using the statistical software R (https://www.r-project.org/)^32^.

Teeth from killer whales that stranded in ECA (n = 10 individuals) and NWA (n = 7) (Fig. 1) and mandibular bone from killer whales that stranded in Greenland (n = 2) and Denmark (n = 3) were acquired from government and museum collections (Table 1). Of the 10 ECA whales, nine either live-stranded or were found frozen in minimal state of decomposition; one had no stranding information. Of these 10 animals, two groups of three whales stranded together, while the other four were alone (Table 1). Of the seven NWA whales, five either live-stranded or were found in minimal state of decomposition, one was found in an advanced state of decomposition, and one had no information. Of these seven animals, two groups of two whales stranded together, while the other three were alone (Table 1).

**Table 1:** Information on tooth and bone specimens collected from killer whales that stranded in the eastern Canadian Arctic (ECA) and Northwest Atlantic (NWA), as well as Greenland and Denmark.

Region	Specimen	Other ID	Year	Age (year)	Tissue	Stranding notes
Eastern Canadian Arctic (ECA)	ECA-AB-1948	MM 406	1948	17	Dentine	None available
	ECA-CS-1977-1		1977	4	Dentine	Part of a larger group of 14 killer whales that became entrapped in a freshwater lake and were subsequently killed^2^
	ECA-CS-1977-2		1977	18	Dentine	
	ECA-CS-1977-3		1977		Dentine	
	ECA-BL-1978	5754-26	1978	17	Dentine	Alone; swam inland via river and was subsequently killed^2^
	ECA-RB-2009		2009	28	Dentine	Alone; sampled 1 week after initial sighting; some decomposition
	ECA-CH-2011		2011	35	Dentine	Alone; some decomposition but little bleaching of pigment
	ECA-SQ-2016-1	ARSQxx1397	2016	6	Dentine	Stranded as a group over a ~ 2-week period after overwintering in the area; live sightings confirmed in previous months^33^
	ECA-SQ-2016-2	ARSQxx1379	2016	34	Dentine	
	ECA-SQ-2016-3	KW2	2016	8	Dentine	
Northwest Atlantic (NWA)	NWA-CB-1971-2	D118-71	1971	29	Dentine	Both ‘killed’ on same date at same location
	NWA-CB-1971-1	D119-71	1971	31	Dentine	
	NWA-SC-1975-1	VMK 5	1975	20	Dentine	Stranded at same location and sampled within 4 days of each other; some decomposition (bloating)
	NWA-SC-1975-2	VMK 6	1975	23	Dentine	
	NWA-SI-1977	10,783	1977	13	Dentine	None available
	NWA-BB-2002		2002	3	Dentine	found in an advanced state of decomposition
	NWA-BP-1998		1998	5	Dentine	1 of 2 that live-stranded
Greenland		M1647	2014		Bone	None available
		M1648	2014		Bone	None available
Denmark		CN89	1980		Bone	None available
		MCE 1068	1995		Bone	None available
		M1646	2007		Bone	None available

Four groups of whales stranded together in pairs or groups of three (ECA-CS-1977-X, ECA-SQ-2016-X, NWA-CB-1971-X, and NWA-SC-1975-X), while all others were alone. Most whales were stranded alive or in minimal state of decomposition. Stranding locations are shown in Fig. 1.

Dentine was collected for *δ*^18^O_P_ analysis by micromilling across one half of a longitudinally bisected tooth. Relatively small sample requirements of *δ*^18^O_SC_ analysis also allowed for sampling of individual annual growth layer groups (GLGs) from a subset of ECA (n = 2) and all NWA (n = 7) whales to assess within-tooth *δ*^18^O_SC_ variation. Mandibular bone was sampled from skulls lacking teeth using a handheld rotary tool. Bone should be directly comparable to dentine because the *δ*^18^O_P_ of both tissues varies linearly with *δ*^18^O of environmental water with similar intercepts and slopes^21,22^, and any effect of cooler ambient temperatures of narrow jaws on ^18^O fractionation should be similar for teeth and mandibles^29^. Both whole-tooth and bone samples also integrate long-term isotopic composition, with the caveat that bone is subject to remodelling and dentine generally is not^34^.

### Oxygen and carbon isotope analysis of dentine and bone

All stable isotope analyses were performed at the Laboratory for Stable Isotope Science (LSIS) at the University of Western Ontario, London, Ontario, Canada. Dentine and bone *δ*^18^O_P_ and *δ*^18^O_SC_ were analyzed following procedures described in Matthews et al.^22^. Briefly, for isotopic analysis of phosphate oxygen, powdered samples (25 to 35 mg) were dissolved in 3 M acetic acid, and silver phosphate (Ag_3_PO_4_) was then precipitated following the ammonia volatilization method^35,36^. Aliquots of powdered Ag_3_PO_4_ (~ 0.2 mg) were then weighed into silver capsules and introduced into a Thermo Scientific™ High Temperature Conversion Elemental Analyzer (TC/EA). The resulting carbon monoxide (CO) gas was purified on a GC column packed with a 5 Å molecular sieve and swept in a continuous flow of helium to a Thermo Scientific™ Delta^PLUS^XL™ isotope-ratio mass spectrometer (IRMS) for isotopic analysis.

Phosphate oxygen isotope ratios are reported in *δ*-notation relative to Vienna Standard Mean Ocean Water (VSMOW), calibrated using accepted values for standards IAEA-CH-6 (+ 36.40‰)^37^ and Aldrich Silver Phosphate–98%, Batch 03610EH (+ 11.2‰)^38^. The precision (SD) of these standard analyses ranged from 0.33‰ (IAEA-CH-6, n = 15) to 0.42‰ (Aldrich, n = 40), respectively. The accuracy of this calibration curve was tested using NBS 120c phosphate rock; 11 analyses returned an average *δ*^18^O_P_ =  + 21.57 ± 0.29‰, which compares well with its accepted value of + 21.7‰^39^. The average reproducibility for replicate *δ*^18^O_P_ analyses of samples was ± 0.22‰ (n = 13).

Structural carbonate oxygen and stable carbon isotopes were measured in untreated dentine since the effects of treatment to remove secondary carbonate and organic matter can be inconsistent^22,40,41^. Powdered samples (~ 0.8 mg) were dried overnight at 80 °C in a reaction vial, which was then septa-sealed, capped, and evacuated using an automated sampler (Micromass MultiPrep). Ortho-phosphoric acid was then added to generate carbon dioxide gas (CO_2_) by reaction at 90 °C for 20 min. The evolved CO_2_ was cryogenically scrubbed and transferred to an IRMS (Fisons Optima) for analysis in dual-inlet mode.

*δ*^18^O_SC_ values were calibrated relative to VSMOW using accepted values for NBS 19 (calcite; + 28.65‰) and NBS 18 (calcite; + 7.20‰), with a precision (SD) of 0.05‰ (n = 51) and 0.12‰ (n = 27), respectively. Accuracy and precision (SD) were assessed using laboratory reference materials not included in the calibration curve: WS-1 (calcite; *δ*^18^O measured =  + 26.22 ± 0.22‰, n = 28; accepted =  + 26.23‰), and Suprapur (calcite; *δ*^18^O measured =  + 13.26 ± 0.13‰, n = 24; accepted =  + 13.30‰). *δ*^13^C_SC_ values were calibrated relative to VPDB using accepted values for NBS 19 (calcite; + 1.95‰) and LSVEC (Li-carbonate, − 46.6‰), with a precision (SD) of 0.05‰ (n = 51) and 0.13‰ (n = 24), respectively. Accuracy and precision (SD) were assessed using international and laboratory reference materials not included in the calibration curve: NBS 18 (calcite; *δ*^13^C measured = − 5.08 ± 0.09‰, n = 28; accepted = − 5.01‰); WS-1 (calcite; *δ*^13^C measured =  + 0.76 ± 0.09‰, n = 28; accepted =  + 0.76‰), and Suprapur (calcite; *δ*^13^C measured = − 35.64 ± 0.11‰, n = 24; accepted = − 35.55‰). The average reproducibility for replicate analyses of samples was ± 0.14‰ (n = 16) for *δ*^18^O_SC_ and ± 0.07‰ (n = 16) for *δ*^13^C_SC_.

### Data analysis

We first tested for temporal baseline isotope shifts (e.g., refs.^42,43^) since specimens were collected over an ~ 70-year timespan. Whale ages were estimated from counts of GLGs observed under reflected light, and the median of three counts conducted by one reader (CJDM) over several weeks was taken as the age estimate (Table 1). Calendar year representing the mid-point of tooth deposition was then estimated by subtracting half the whale age from year of death. Linear regression of *δ*^18^O_P_ against calendar year showed no evidence of temporal baseline shifts in isotopic composition over the timeframe (Figure S1). Values of *δ*^13^C_SC_, however, exhibited an apparent linear decline over the same period, although this relationship was not significant (Figure S2). However, the estimated slope (− 0.023‰ year^−1^) was similar in direction and magnitude to declines in North Atlantic *δ*^13^C attributed to the Suess effect^42,43^. *δ*^13^C_SC_ values were therefore adjusted to the most recent sample year (2013) by subtracting 0.023‰ year^−1^ to remove temporal bias.

Differences in *δ*^18^O_P_ among groups based on stranding location were assessed using the non-parametric Kruskal–Wallis rank sum test, since residuals from one-way ANOVAs violated assumptions of normality and heteroscedasticity. In addition to the ECA and NWA killer whales, we included data from ECA beluga whales and Gulf of Mexico (GoM) dolphins^22^ (Table S1) as *δ*^18^O reference ‘endpoints’, as they are from regions representing extreme low and high ends, respectively, of the *δ*^18^O range across the North Atlantic. We assume any biases stemming from different body sizes or diets of the species are minimal, given the relatively small deviations (< 1‰) of dentine/bone *δ*^18^O of a large number of cetacean species from linear relationships with environmental *δ*^18^O^21,22^. Although the two GoM dolphin species, common bottlenose dolphins (*Tursiops truncatus*) and Atlantic spotted dolphins (*Stenella frontalis*), exhibit nearshore-offshore habitat partitioning along a gradient in surface water *δ*^18^O values^23^, their *δ*^18^O_P_ were statistically indistinguishable^22^ and therefore grouped in our analyses. Greenland and Denmark killer whales were excluded from these analyses given their small sample sizes. Significant Kruskal–Wallis tests were followed by Dunn’s multiple comparison *post-hoc* test with Benjamini–Hochberg adjustment of *p*-values^44^. All analyses were done using R version 4.0.0^32^ and its associated package ‘FSA’^45^.

Hierarchical cluster analysis of *δ*^18^O_P_ based on a Euclidean distances and average linkage was performed on all killer whales (including Greenland and Denmark samples) using the R packages ‘cluster’^46^ and ‘dendextend’^47^.

Geographic distributions were assigned by first estimating source *δ*^18^O_marine_ from each individual killer whale’s dentine *δ*^18^O_P_ by rearranging the following published calibration:

1 $$\delta^{18} {\text{O}}_{{\text{P}}} = \,\upbeta _{0} +\upbeta _{1} \times \delta^{18} {\text{O}}_{{{\text{marine}}}} ,$$

where the intercept (β_0_, 18.73 ± 0.30‰) and slope (β_1_, 0.81 ± 0.23‰) estimates are based largely on dentine *δ*^18^O_P_ measurements of cetaceans from the North Atlantic and associated basins with heterogenous *δ*^18^O_marine_^22^. Intercept and slope standard deviations and *δ*^18^O_P_ measurement error were propagated through to final *δ*^18^O_marine_ estimates using first-order Taylor series expansion in the R package ‘propagate’^48^. *δ*^18^O_marine_ values encompassed by the estimated 95% CI for each individual killer whale were then extracted from modelled North Atlantic gridded surface *δ*^18^O data^23^ downloaded from Global Seawater Oxygen-18 Database^49^. Marine *δ*^13^C isoscapes^20^ were not used in this manner because confounding dietary and metabolic influences on dentine *δ*^13^C_SC_ values^50^ could not be constrained to allow for direct comparison with baseline DIC or phytoplankton values.

Finally, the standard deviation of individual GLG *δ*^18^O_SC_ measurements was calculated as an index of within-tooth variation for each of the 9 teeth that were measured.

### Ethics approval

Ethics approval was not required for museum specimens used in this study. Destructive sampling was formally approved through museum protocols.

## Results

**Figure 2:**
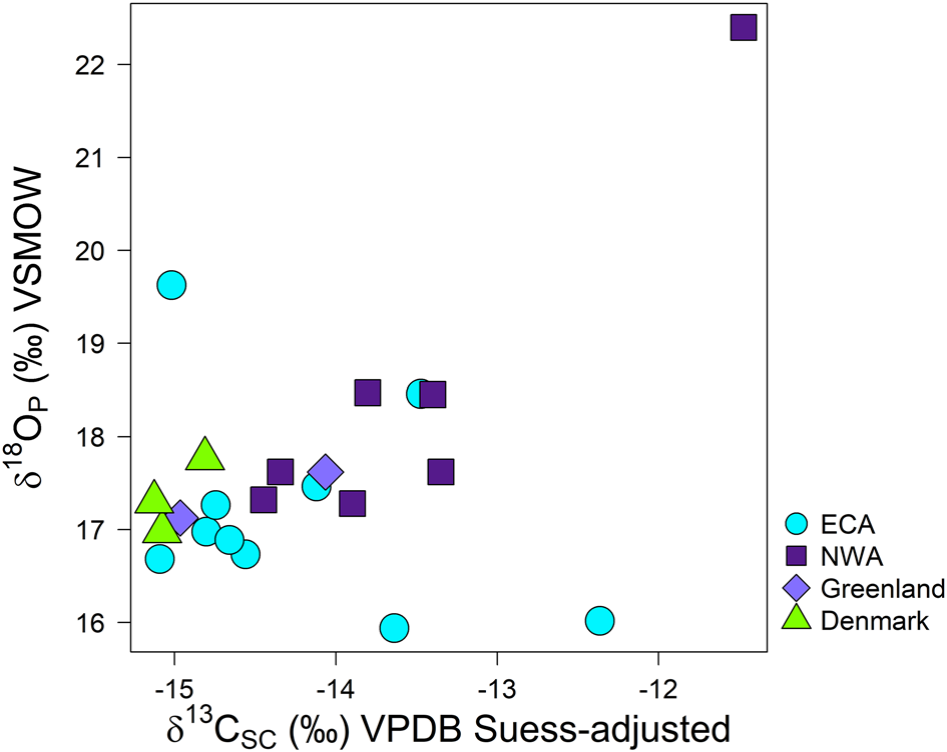
Biplot of dentine *δ*^18^O_P_ versus Suess-adjusted *δ*^13^C_SC_ values of teeth from eastern Canadian Arctic (ECA; turquoise circles) and Northwest Atlantic (NWA; purple squares) killer whales relative to bone values of killer whales from Greenland (light purple diamonds) and Denmark (green triangles). The range of *δ*^18^O_P_ indicates considerable spatial separation. This result reflects latitudinal gradients in the North Atlantic, suggesting considerable spatial structuring among killer whales in both the ECA and NWA. Although dietary influences on *δ*^13^C confound direct interpretations within a spatial context, the range in *δ*^13^C_SC_ values exceeds that expected due to dietary differences alone. The *δ*^13^C_SC_ were adjusted by − 0.023‰ year^−1^ up to the year 2013 (the most recent sample) to account for temporal bias due to baseline shifts (Suess effect^42,43^) in North Atlantic *δ*^13^C over the timespan of collected specimens (see Figure S2).

Individual killer whale dentine *δ*^18^O_P_ values ranged from + 15.94 to + 22.40‰ (Table 2, Fig. 2). Mean *δ*^18^O_P_ values differed among species/regions (Kruskal–Wallis, Chi square = 16.2, *p* = 0.0010, df = 3), although pairwise comparisons indicated whales that stranded in the ECA (+ 17.21 ± 1.11‰) and NWA (+ 18.45 ± 1.81‰) were not significantly different (*p* = 0.087). ECA killer whale *δ*^18^O_P_ did not differ from ECA belugas (+ 17.38 ± 0.69‰, *p* = 0.48) but was significantly lower than GoM dolphins (+ 18.82 ± 0.86‰, *p* = 0.0027) (Table S1). The *δ*^18^O_P_ of NWA whales did not differ from ECA belugas (*p* = 0.14) or GoM dolphins (*p* = 0.23). Mean bone *δ*^18^O_P_ values of the Greenland and Denmark killer whales were + 17.4 ± 0.3‰ and + 17.4 ± 0.4‰, respectively (Table 2, Fig. 2). Suess-adjusted *δ*^13^C_SC_ values ranged from − 15.1 to − 11.5‰ across all whales (Fig. 2).

**Table 2:** ‘Whole-tooth’ dentine *δ*^18^O_P_ and *δ*^13^C_SC_ of killer whales that stranded in the eastern Canadian Arctic (ECA) and Northwest Atlantic (NWA), along with values measured in bone of killer whales from Greenland and Denmark.

Region	Specimen	Tissue	*δ*^18^O_P_ ‰ VSMOW	*δ*^18^O_marine_ ‰ VSMOW	*δ*^13^C_SC_ ‰ VPDB	*δ*^13^C_SC_ ‰ VPDB (Suess-adjusted)
Eastern Canadian Arctic (ECA)	ECA-AB-1948	Dentine	** + 16.89**	− 2.27 ± 0.79	− **12.97**	− **14.66**
	ECA-CS-1977-1	Dentine	+ 16.02	− 3.35 ± 1.06	− 11.49	− 12.36
	ECA-CS-1977-2	Dentine	** + 17.27**	− 1.80 ± 0.69	**13.71**	− **14.75**
	ECA-CS-1977-3	Dentine	** + 19.63**	+ 1.11 ± 0.56	− 14.19	− 15.02
	ECA-BL-1978*	Dentine	** + 17.47**	− 1.56 ± 0.64	− **13.12**	− **14.12**
	ECA-RB-2009*^+^	Dentine	+ 18.46	− 0.33 ± 0.47	− **13.06**	− **13.47**
	ECA-CH-2011	Dentine	+ 15.94	− 3.44 ± 1.08	− **13.19**	− **13.64**
	ECA-SQ-2016-1	Dentine	** + 16.74**	− 2.46 ± 0.84	− 14.56	− 14.56
	ECA-SQ-2016-2	Dentine	** + 16.69**	− 2.52 ± 0.85	− 14.77	− 15.09
	ECA-SQ-2016-3	Dentine	** + 16.98**	− 2.16 ± 0.77	− 14.78	− 14.80
Mean ± SD			+ 17.21 ± 1.11		− 13.58 ± 1.03	− 14.25 ± 1.03
Northwest Atlantic (NWA)	NWA-CB-1971-2*	Dentine	** + 18.45**	− 0.35 ± 0.47	− 12.10	− 13.40
	NWA-CB-1971-1*	Dentine	+ 18.47	− 0.32 ± 0.47	− **12.48**	− **13.80**
	NWA-SC-1975-1*	Dentine	+ 17.62	− 1.37 ± 0.60	− 13.24	− 14.34
	NWA-SC-1975-2*	Dentine	+ 17.62	− 1.37 ± 0.60	− **12.21**	− **13.35**
	NWA-SI-1977*	Dentine	+ 17.32	− 1.74 ± 0.67	− **13.47**	− **14.45**
	NWA-BB-2002*	Dentine	+ 17.28	− 1.79 ± 0.69	− **13.61**	− **11.47**
	NWA-BP-1998*^+^	Dentine	** + 22.40**	+ 4.53 ± 1.37	− **11.07**	− **13.90**
Mean ± SD			+ 18.45 ± 1.81		− 12.60 ± 0.91	− 13.53 ± 0.91
Greenland		Bone	** + 17.62**	− 1.37 ± 0.60	− 13.80	− 14.96
		Bone	** + 17.13**	− 1.98 ± 0.72	− **14.70**	− 14.06
Mean ± SD			+ 17.37 ± 0.35		− 14.51 ± 0.64	− 14.25 ± 0.64
Denmark		Bone	** + 16.98**	− 2.16 ± 0.77	− 14.03	− 15.8
		Bone	** + 17.77**	− 1.19 ± 0.57	− 14.11	− 14.81
		Bone	+ 17.30	− 1.77 ± 0.68	− 14.70	− 15.13
Mean ± SD			+ 17.35 ± 0.39		− 14.28 ± 0.37	− 15.00 ± 0.37

Source marine values (*δ*^18^O_marine_) were estimated from a published calibration^22^ and form the basis of the inferred geographic distributions presented in Fig. 4. Suess-adjusted *δ*^13^C_SC_ values account for temporal bias due to baseline shifts in North Atlantic *δ*^13^C (see Figure S2) by adding − 0.023‰ year^−1^ * (2013—sample year—half the whale’s age) to *δ*^13^C_SC_. Values shown in bold are the averages of replicate analyses.

*Annual dentine growth layer groups (GLGs) of these whales were also measured for *δ*^18^O in structural carbonate (see Fig. 5).

^+^These whales have previously identified differences in diet and morphology^51,52^.

**Figure 3:**
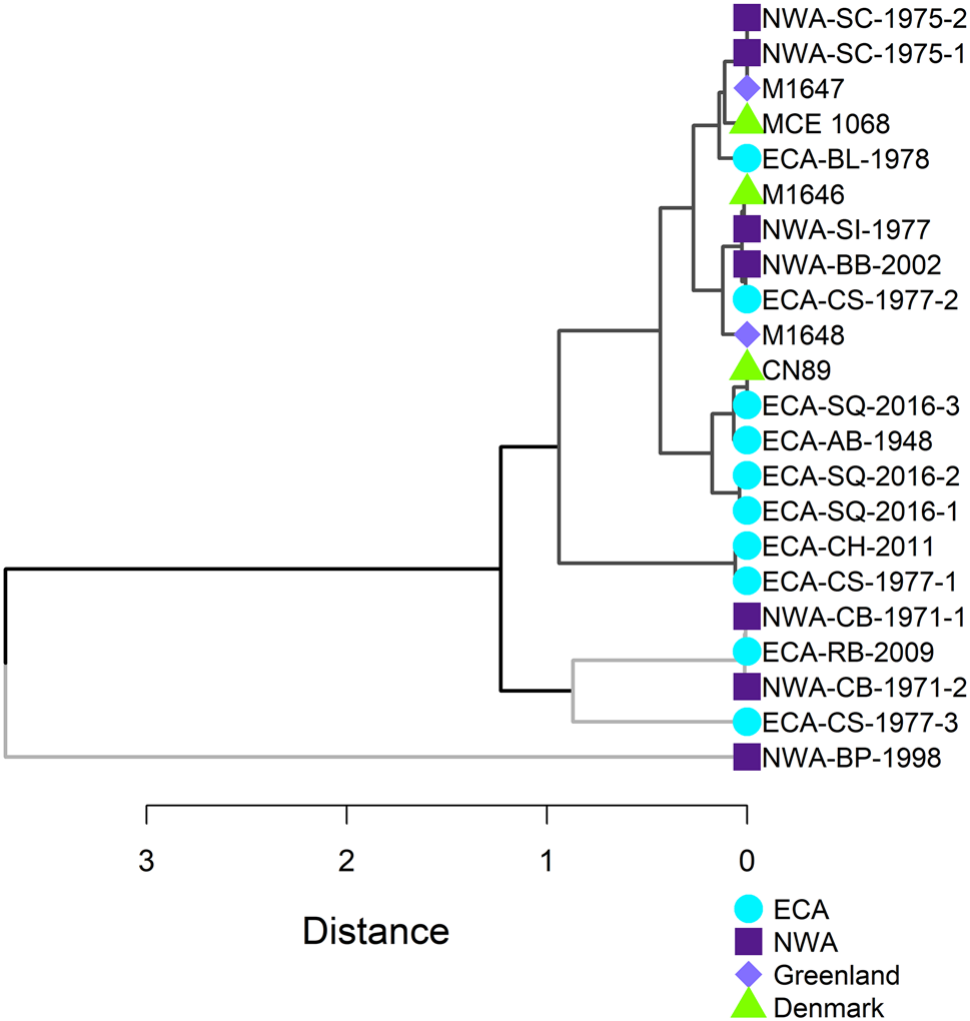
Hierarchical cluster analysis of killer whale dentine *δ*^18^O_P_ based on Euclidean distances and average linkage produced clusters that do not align with stranding locations in the eastern Canadian Arctic (ECA) and northwest Atlantic (NWA). The two most distant (bottom) clusters comprising five whales, indicated with light grey branches, had higher *δ*^18^O_P_ and broader inferred distributions at lower latitudes than whales indicated by dark grey branches (see Fig. 4).

Cluster analysis of *δ*^18^O_P_ produced groupings that were not consistent with stranding location. The most distant clusters comprised five killer whales from both the ECA and NWA with the highest *δ*^18^O_P_ values (Fig. 3). The most distant whale, NWA-BP-1998, had a *δ*^18^O value that was 2 to 6‰ higher than the rest of the sampled whales (Fig. 3). The remaining clusters comprised killer whales that stranded in the ECA, NWA, Greenland, and Denmark, with no clear patterns or divisions based on stranding region within them (Fig. 3). Most whales that stranded together clustered together with similar *δ*^18^O_P_ (whales NWA-SC-1975-1 and NWA-SC-1975-2; whales ECA-SQ-2016-1 through 3; and whales NWA-CB-1971-1 and NWA-CB-1971-2); the exception was the three whales that stranded together in Cumberland Sound in 1977 (the ECA-CS-1977 series) (Fig. 3).

**Figure 4:**
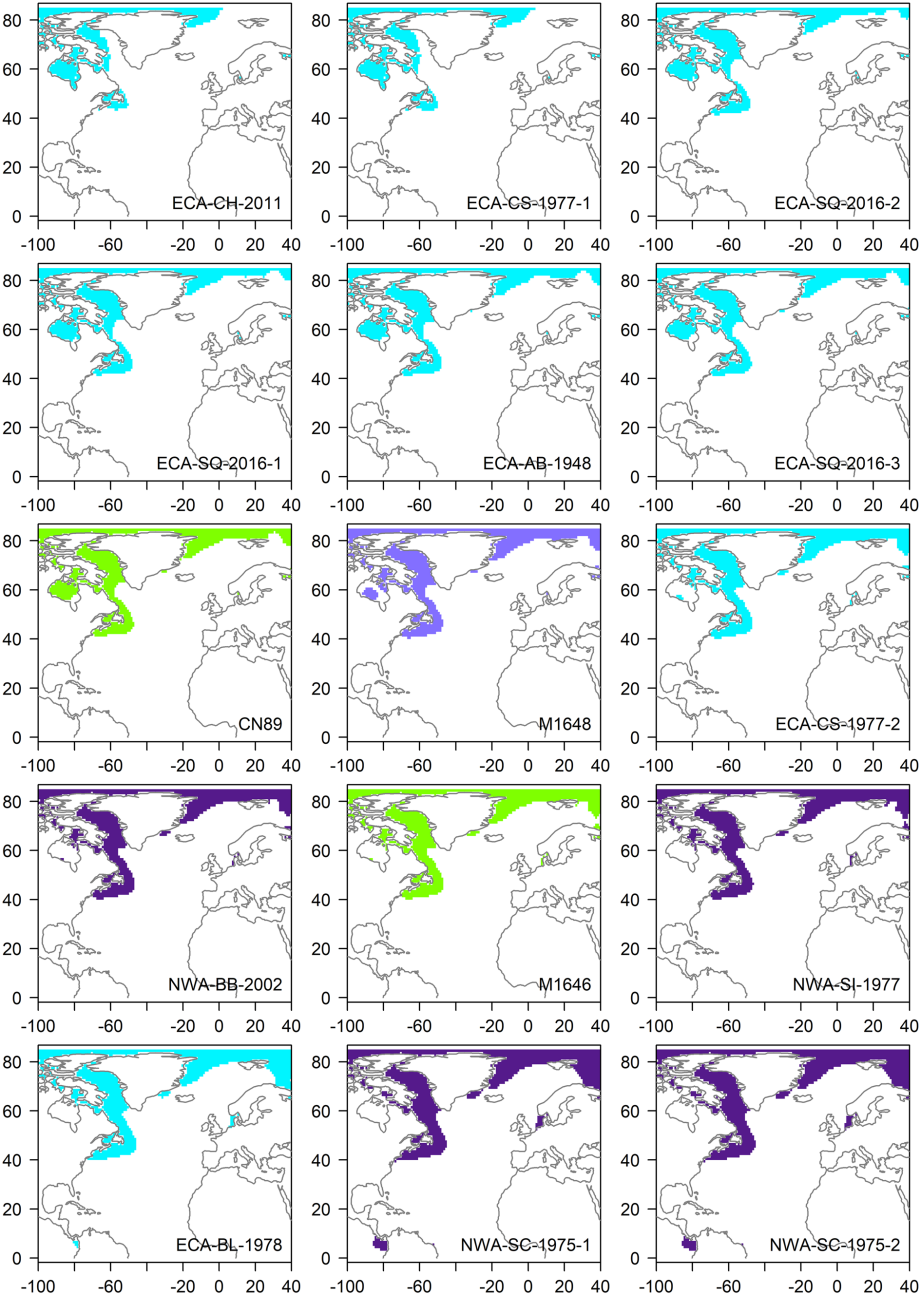

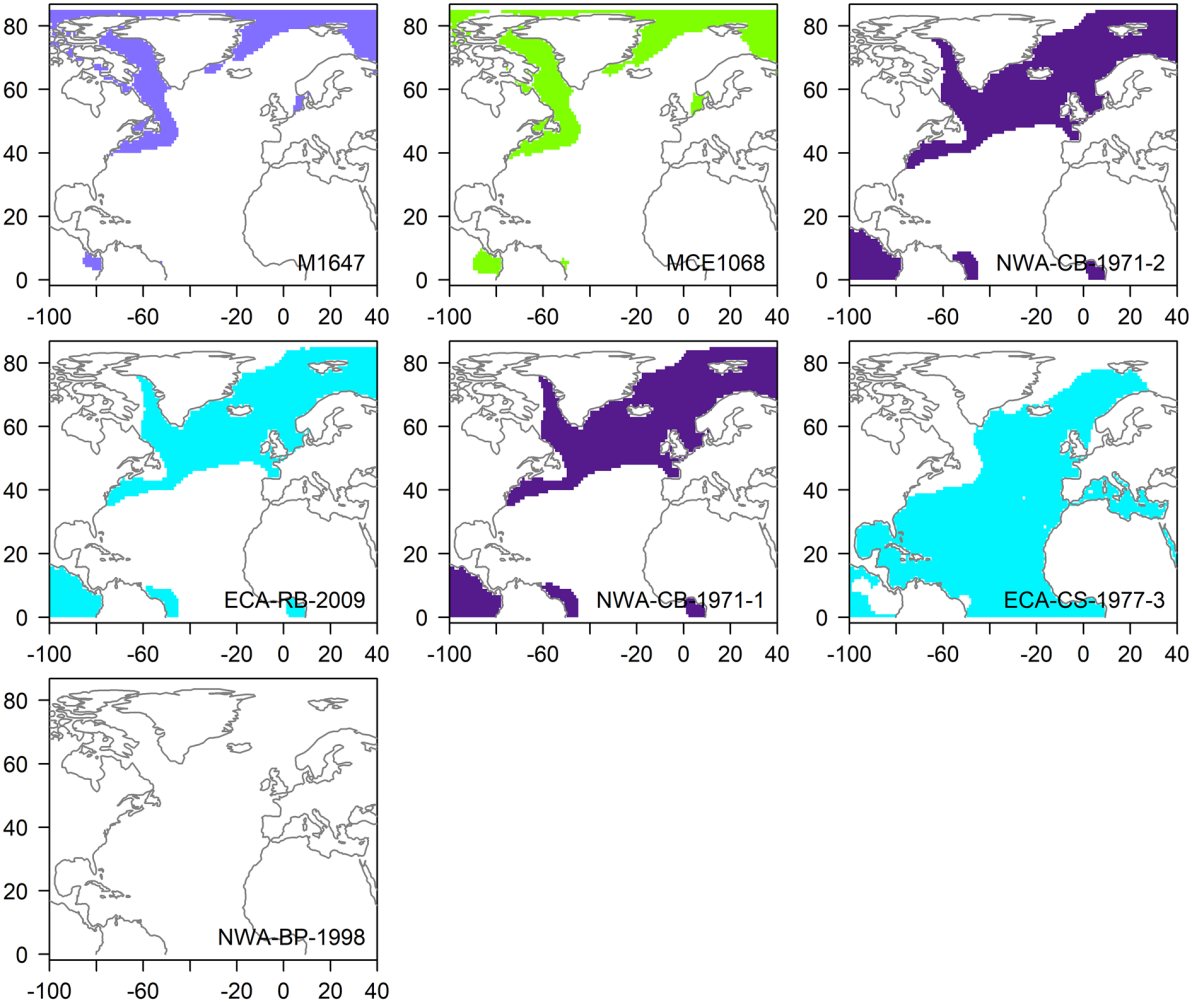
Inferred geographic distributions of killer whales that stranded in the Eastern Canadian Arctic (ECA; turquoise), the Northwest Atlantic (NWA; purple), Greenland (light purple), and Denmark (green). Individual maps are presented in increasing order of measured *δ*^18^O_P_. The map for whale NWA-BP-1998 is empty because its predicted *δ*^18^O_marine_ exceeded predicted isoscape values. Distributions were drawn by extracting values from the gridded surface *δ*^18^O data (downloaded from the Global Seawater Oxygen-18 Database; https://data.giss.nasa.gov) that correspond to the 95% CI of source marine δ^18^O estimated from dentine δ18OP (see Table 2). Maps were created using R version 4.0.0^32^ and associated packages ‘sp’^53,54^, ‘raster’^55^, ‘ncdf4′^56^, and ‘rgdal’^57^. The continents shapefile was downloaded from Natural Earth (https://www.naturalearthdata.com).

Estimated source *δ*^18^O_marine_ values of ECA/NWA whales ranged from − 3.44‰ to + 4.53‰ (Table 2). The estimated 95% CI of most whales (n = 13) corresponded to high-latitude ^18^O-depleted waters characteristic of coastal ECA, NL, and Greenland. Several killer whales (n = 5), however, had inferred distributions that largely excluded high-latitude coastal areas and encompassed a broader band of the North Atlantic, including the relatively ^18^O-enriched waters south of the Gulf Stream (Fig. 4). The 95% CI around predicted source *δ*^18^O_marine_ values of whale NWA-BP-1998 completely exceeded predicted isoscape values (Fig. 4). Estimated source *δ*^18^O_marine_ values of the Greenland and Denmark killer whales (Table 2) also corresponded to high-latitude ^18^O-depleted waters (Fig. 4).

**Figure 5:**
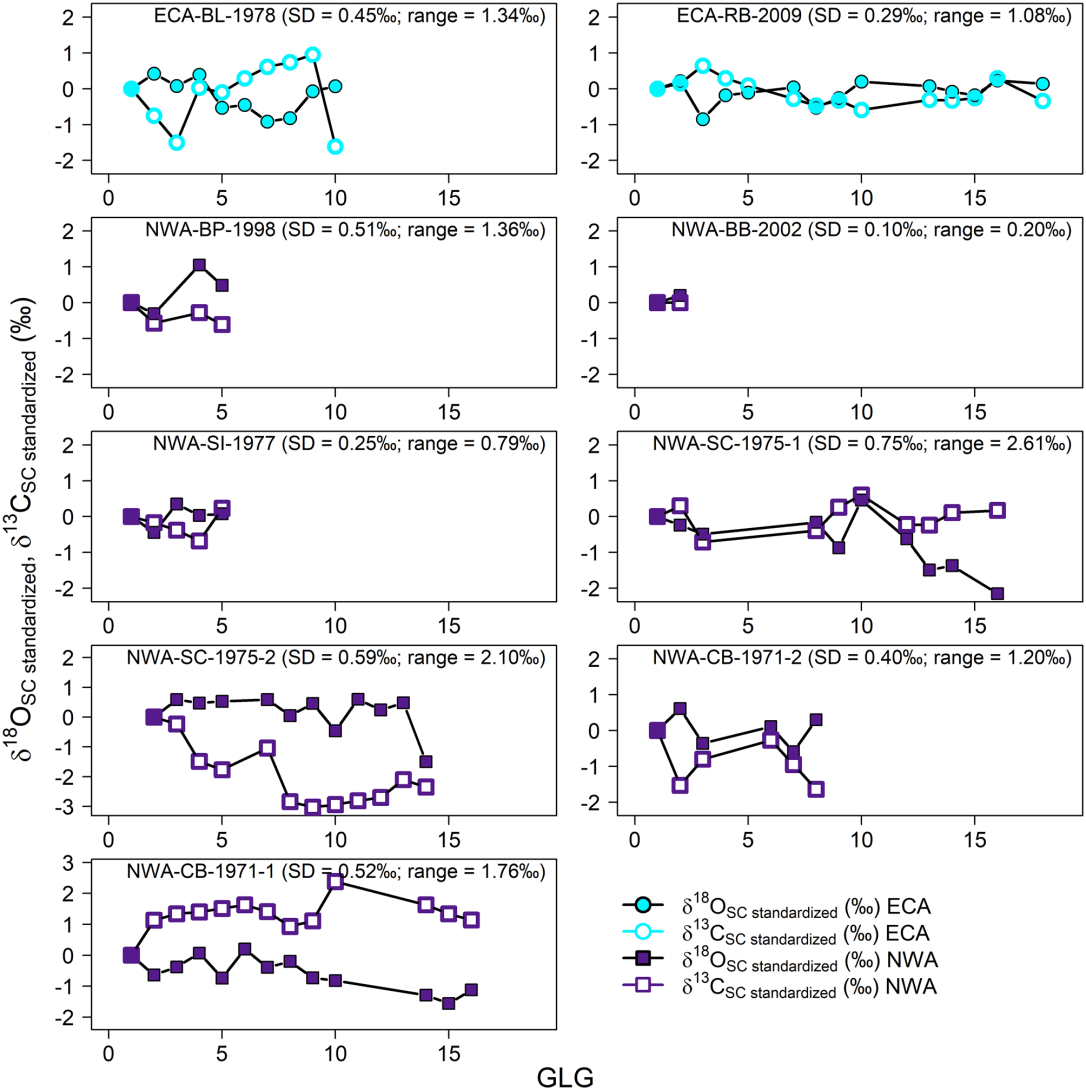
*δ*^18^O_SC_ (solid) and Suess-adjusted *δ*^13^C_SC_ (open) of individual annual dentine growth layer groups (GLGs) of teeth from killer whales that stranded in the eastern Canadian Arctic (ECA; turquoise circles) and the Northwest Atlantic (NWA; purple squares). Values are standardized to that of the first GLG. Higher within-tooth *δ*^18^O_SC_ variation indicates greater interannual variability in distribution. Although dietary influences on *δ*^13^C_SC_ cannot be constrained, concurrent (often inverse) variation in *δ*^13^C_SC_ with *δ*^18^O_SC_ in most of the profiles is consistent with patterns exhibited in the North Atlantic *δ*^18^O and *δ*^13^C isoscapes^19^. Note different axes of two lower left panels.

The range of dentine *δ*^18^O_SC_ values within the subset of nine teeth averaged 1.36‰, and was lowest in whale NWA-BB-2002 (0.20‰—although this whale’s profile comprised only two data points) and highest in whale NWA-SC-1975-1 (2.61‰) (Fig. 5). Within-tooth *δ*^18^O_SC_ variation was generally greater in NWA than ECA killer whales (Fig. 5). *δ*^18^O_SC_ variation was typically accompanied by concurrent, often inverse, variation in Suess-adjusted *δ*^13^C_SC_ (Fig. 5).

## Discussion

Distribution patterns have not been fully described for even the best-studied killer whale populations (e.g., ref.^58^), as observational effort has been limited primarily to nearshore areas when killer whales are seasonally abundant (e.g., ref.^59^), and photo-ID catalogues have not been developed for most regions. Our assessment of killer whale distributions using dentine oxygen isotopes integrated over long timeframes has revealed patterns in the species’ distribution in the North Atlantic that have been suggested, but not resolved, by previous sightings compilations across the region^2–4,6,14,60^. The ranges of most whales were restricted to high-latitude ^18^O-depleted waters; however, the broader ranges of a smaller number of whales encompassing lower latitude ^18^O-enriched waters in the North Atlantic indicates a considerable degree of spatial structure among killer whales in the ECA and NWA that has not been reported previously.

Isoscapes have been used routinely to infer distributions and movements of terrestrial animals^18^, but their use in marine systems has been hindered by limited baseline measurements over appropriate spatiotemporal scales^20,61^. LeGrande and Schmidt’s^23^ predicted gridded marine *δ*^18^O isoscape was constructed using a 50-year dataset combined with modeling of ^18^O-salinity relationships, and the spatiotemporal coverage of samples used to inform and validate their model is considered to be relatively good in the Arctic and North Atlantic Oceans. However, there are areas of sparse coverage and the authors caution that their use of multi-seasonal, multi-decadal data would obscure variation in Arctic surface water *δ*^18^O, which is temporally and spatially more variable than ice-free waters due to seasonal cycles of ice formation and melt^62–64^. Discrepancy between estimated source *δ*^18^O_marine_ values and modeled isoscape values could also reflect the fact that only variance in predicted source *δ*^18^O_marine_ values was incorporated in our assignment of distributions (via 95% CIs), while variance in predicted isoscape values was not. The much greater discrepancy observed for whale NWA-BP-1998, for which only the lower limits of the 99% CI of its predicted source *δ*^18^O_marine_ corresponded to isoscape values in lower latitudes of the North Atlantic (not shown), could reflect extremely sparse sample coverage informing modeled values in that region^23^.

The most obvious pattern in our data is the spread in *δ*^18^O_P_ values and estimated *δ*^18^O_marine_ values, which encompass both sides of the sharp boundary in marine *δ*^18^O values demarcated by the Gulf Stream^23^. The inferred high-latitude distributions of most killer whales above this boundary is consistent with global patterns that show killer whale density increases by 1–2 orders of magnitude from tropical to polar regions^1^, including in the NWA, where comparatively few killer whale records exist from the eastern seaboard of the U.S.A. south to the equator^4^. However, inferred distributions of several killer whales that stranded in both the ECA and NWA encompassed relatively ^18^O-enriched, lower latitude waters across a broader area of the North Atlantic. We can rule out passive transport of carcasses from distant locations via currents as a source of variation for these five whales, which either live-stranded (ECA-CS-1977, NWA-CB-1971-1 and 2, and NWA-BP-1998) or were found in inland Arctic waters in minimal state of decomposition (ECA-RB-2009). Whales observed seasonally in the ECA have been hypothesized to range possibly as far south as the Caribbean during winter^2^, and recent studies have demonstrated^10^ or inferred^17^ movements of ECA killer whales into temperate and perhaps tropical waters. Killer whales are observed year-round in low numbers throughout the Caribbean^4,65^, and large numbers of killer whales were observed by whalers in the 1800s in a widespread offshore area known to them as the Western Ground, which stretched from Bermuda to the Azores^14^. Limited data preclude interpretations of killer whale residence patterns in either region^4,65^, although Katona et al.^4^ suggested there could be resident and migratory populations stretching from the Bay of Fundy to the equator.

The largely similar inferred distributions of most of the killer whales in the high-latitude waters surrounding the ECA, NL, and Greenland could reflect a continuous distribution across the region, as has been hypothesized^2^. However, the ~ 2‰ range of individual *δ*^18^O_P_ values among these whales exceeds the degree of variability expected for cetacean populations with similar distributions within waters with homogenous *δ*^18^O. Clementz and Koch^66^ reported *δ*^18^O ranges of ~ 0.5 to 1‰ in tooth enamel of cetacean populations from the isotopically homogenous Californian coast. For comparison with other North Atlantic cetaceans, bone carbonate *δ*^18^O differed by just 0.4‰ between fin whales (*Balaenoptera physalus*) from western Iceland and northwestern Spain^31^, and dentine carbonate *δ*^18^O differed by a similar amount between sperm whales (*Physeter macrocephalus*) from northwestern Spain and Denmark^30^. While groupings based on stranding location did not differ significantly, spatial differences among individuals inferred from *δ*^18^O_P_ are largely consistent with previous bulk and amino acid-specific *δ*^15^N analyses that suggested some degree of spatial separation among these same killer whales^51^.

Geographic assignments can be refined by using multiple isotopes to narrow potential distributions to areas where tissue values correspond with intersecting isoscape values^67,68^. Carbonate in biogenic apatite precipitates from blood bicarbonate and *δ*^13^C_SC_ thus reflects the bulk isotopic composition of respired carbohydrates, lipids and proteins^49,69,70^. Despite advances in modelling spatial variation in DIC and phytoplankton *δ*^13^C across the North Atlantic^20^, calibration of killer whale dentine *δ*^13^C_SC_ values to the predicted *δ*^13^C isoscape would therefore require knowledge not only of trophic position, but also the fraction of carbon from dietary lipids and proteins routed to *δ*^13^C_SC_ (see ref.^66^). However, the ~ 4‰ range in *δ*^13^C_SC_ among killer whales exceeds that expected from trophic factors alone, particularly since most of the killer whales in our sample fed at a similar tropic position^51,52^. With more detailed dietary information to allow for partitioning of trophic and spatial influences on *δ*^13^C_SC_, relative *δ*^13^C_SC_ differences among killer whales with similar *δ*^18^O_P_ values could offer additional discriminatory power, particularly in areas of uniformly ^18^O-depleted coastal, high latitude waters.

Myrick et al.^71^ demonstrated a constant rate of dentine deposition across all months in a captive killer whale, which we assume is also true of wild killer whales. However, dentine deposition rates vary seasonally in many odontocete species in response to seasonal food availability or environmental variation, with more material typically laid down from spring to fall^72,73^. If this deposition pattern occurs in wild killer whales, then our analysis of whole-tooth samples and subsequent inferences about distributions would be biased towards summer months. Bone, however, is slowly and continually remodelled^34^; as such, bone *δ*^18^O_P_ of the five whales from Greenland and Denmark reflect a long-term average distribution within high-latitude, ^18^O-depleted waters. That being said, temporally crude whole-tooth and bone samples would obscure any seasonal movements^10^ or interannual variation in distribution. Within-tooth variation in *δ*^18^O_SC_ on the order of 1–2‰ suggests interannual variation in ECA and NWA killer whale distributions. By comparison, dentine carbonate *δ*^18^O declines of ~ 2‰ across male North Atlantic sperm whale teeth were linked possibly to large-scale latitudinal migrations at sexual maturity^30^. Large-scale movements of killer whales have now been documented globally, including the North Atlantic^10^. However, their ranging patterns remain poorly understood, and fine-scale spatial resolution (i.e., *µ*m) afforded by in situ microbeam sampling techniques^74^ could help resolve seasonal and interannual variation in movements and distribution.

Spatial structure inferred here from oxygen isotopes is supported, at least generally, by previous studies indicating genetic differentiation among ECA and NWA killer whales. Foote et al.^75^, using samples not included in this study, showed NWA whales formed a different clade than other North Atlantic killer whales. In a more recent analysis that sequenced whole genomes, Lefort^76^ differentiated two clusters among ECA and NWA killer whales: one that comprised animals from northern Baffin Island and Newfoundland, and another that comprised animals from Hudson Bay and Baffin Island. There was also high covariance among killer whales from the low Arctic (Hudson Bay) and those from the eastern North Atlantic (Greenland, Iceland, and Norway)^76^, which is consistent with isotope clusters that comprised animals from the ECA and Greenland and Denmark. We note, however, that the Hudson Bay whale included in Lefort^76^, ECA-RB-2009, was relatively distant from the five Greenland and Denmark specimens based on *δ*^18^O_P_ values. Of the four animals included in both studies, low genetic variation was observed between whales ECA-AB-1948 and ECA-RB-2009 and between whales NWA-CB-1971-2 and NWA-BB-2002^76^. Neither of these pairings, however, clustered tightly together based on *δ*^18^O_P_ (Fig. 3).

Oxygen isotope data are consistent with purported killer whale ecotypes in the ECA and NWA^52^. Two of the killer whales with inferred broader, lower latitude distributions (ECA-RB-2009 and NWA-BP-1998) have amino acid-specific *δ*^15^N patterns indicative of dietary differences from the other whales, coupled with morphological differences in tooth wear^51,52^. These whales appear to be ecologically and morphologically similar to the North Atlantic Type 1 killer whales that range from NL to Norway^75,77^, and morphologically similar to a single killer whale specimen from the Caribbean^65^. We assume that distribution, and not dietary, differences are the primary driver of observed *δ*^18^O_P_ variation among these whales, as ingestion of water via food and metabolic water formed via oxidation of food are thought to be relatively minor oxygen fluxes compared to diffusion of water across cetacean skin^24,25^. This assumption is supported by similar dentine *δ*^18^O_P_ values of fish-eating and mammal-eating killer whale ecotypes from the eastern North Pacific (16.75 vs. 16.87‰, respectively)^22^. It is also supported more generally by relatively small deviations (< 1‰) of dentine/bone *δ*^18^O of whale species with varied diets from linear relationships with environmental *δ*^18^O^21,22^. Vertical variation in *δ*^18^O_marine_ could potentially confound our interpretations, if, for example, higher killer whale oxygen isotope compositions were acquired while spending significant amounts of time foraging in deep-water on species such as Greenland shark (*Somniosus microcephalus*). Greenland sharks from northern Baffin Island have relatively ^18^O-enriched tooth phosphate that corresponds to *δ*^18^O_marine_ at depths exceeding 300 to 500 m^78^. While this depth is within the dive range of killer whales^79^, the ~ 6‰ range in *δ*^18^O_P_ separating the sampled killer whales (Fig. 2) exceeds the ~ 1‰ vertical increase in *δ*^18^O_marine_ from 0–250 m (~ − 1.5‰) to > 250 m depths (~ − 0.5‰) in Baffin Bay^62^.

The oxygen isotope compositions of groups of killer whales that stranded together also allow for some inference on the nature of their associations. Similar *δ*^18^O_P_ values among individuals of three of the four groups of animals that stranded together indicate they likely comprised stable, long-term associations with similar distribution and movement history, consistent with research indicating offspring generally do not disperse from killer whale matrilines^80^. However, the close to 4‰ spread in *δ*^18^O_P_ among the group of three killer whales that stranded together in Cumberland Sound in 1977 suggests they were not part of a cohesive group. The different ages (and thus sizes) of these whales does not appear to be a factor. While there is some suggestion of a decline in *δ*^18^O_P_ with age in some of the older NWA whales, no such pattern was observed for the ECA whales (Fig. 5). Moreover, the group of three killer whales that stranded together in southeast Hudson Bay (ECA-SQ series) had almost identical *δ*^18^O_P_ despite having a similar age range (Tables 1, 2). The three killer whales from Cumberland Sound were sampled from a group of 14 animals that became entrapped in a saltwater lake while hunting belugas^2^. Temporary associations between small, stable groups of killer whales have been observed in other locations where prey resources are highly localized^81^, a possibility in Cumberland Sound where belugas are seasonally aggregated in a relatively small area.

The high-latitude distributions inferred for most of the sampled whales is relevant for understanding potential killer whale range expansions and/or population increases with reductions in sea ice extent and duration. However, with half of the ECA specimens and most of the NWA specimens in our sample coming from the 1970s or earlier, much of our sample does not encompass the approximately five-decade period over which sightings have increased dramatically in both the ECA and off NL^5,6,11,82^. Distribution shifts in response to changing environmental variables such as declining sea ice extent and duration can be rapid, as exhibited across various taxa in Arctic regions^83^. We therefore recommend further study of factors that drive current killer whale distribution and population structure throughout the North Atlantic, using a range of complementary approaches such as long-term, coarse-scale data reported here versus short-term high-resolution telemetry data^8–10^.

## Supplementary Information

Supplementary Information 1.

Supplementary Information 2.
