# Supplementary material for: Distributions of Arctic and Northwest Atlantic killer whales inferred from oxygen isotopes
Source: Sci Rep. 2021 Mar 24;11:6739. doi: 10.1038/s41598-021-86272-5 (PMC7990931; doi:10.1038/s41598-021-86272-5)
Supplement: Supplementary file 1 — Supplementary Information 1. [file 41598_2021_86272_MOESM1_ESM.docx]

**Distributions of Arctic and Northwest Atlantic killer whales inferred from oxygen isotopes**

**Cory J.D. Matthews^1*^, Fred J. Longstaffe^2^, Jack W. Lawson^3^, Steven H. Ferguson^1^**

*^1^Fisheries and Oceans Canada, 501 University Crescent, Winnipeg, Manitoba, Canada*

*^2^Department of Earth Sciences, The University of Western Ontario, London, Ontario, Canada*

*^3^Fisheries and Oceans Canada, 80 East White Hills Road, St John’s, Newfoundland and Labrador, Canada*

***Correspondence to Cory.Matthews@dfo-mpo.gc.ca

| Table S1: Dentine *δ*^18^O_P_ and *δ*^13^C_SC_ of beluga whales from three eastern Canadian Arctic populations and of dolphins from the Gulf of Mexico presented in Figure 2 and used in statistical analysis of samples based on stranding location. Values shown in **bold** font are the averages of replicate analyses. | | | | | |
| --- | --- | --- | --- | --- | --- |
| Region | Specimen | Year | Tissue | *δ*^18^O_P_ ‰  VSMOW | *δ*^13^C_SC_ ‰ VPDB |
| Canadian Arctic (Eastern High Arctic-Baffin Bay population) | ARGFxx1034 | 2000 | dentine | *+*18.00 | *–*11.99 |
|  | ARGFxx1035 | 2000 | dentine | ***+*18.15** | *–*11.04 |
|  | ARGFxx1044 | 2000 | dentine | *+*16.87 | *–*11.89 |
|  | ARGFxx1032 | 2000 | dentine | *+*18.06 | *–*11.83 |
|  | ARGFxx1040 | 2000 | dentine | *+*18.43 | *–*11.65 |
|  | ARGFxx1059 | 2000 | dentine | *+*17.53 | *–*12.31 |
|  | ARGFxx1036 | 2000 | dentine | *+*18.42 | *–*12.15 |
|  | ARGFxx1033 | 2000 | dentine | *+*17.65 | *–*11.65 |
| Canadian Arctic (Cumberland Sound population) | ARPGxx1040 | 2002 | dentine | ***+*18.43** | ***–*11.65** |
|  | ARPGxx1328 | 2007 | dentine | *+*17.31 | ***–*12.60** |
|  | ARPGxx1317 | 2007 | dentine | *+*16.96 | *–*12.48 |
|  | ARPGxx1226 | 2005 | dentine | *+*17.94 | *–*11.50 |
|  | ARPGxx1034 | 2002 | dentine | *+*17.40 | *–*12.21 |
|  | ARPGxx1232 | 2005 | dentine | *+*16.79 | *–*12.27 |
|  | ARPGxx1037 | 2002 | dentine | *+*17.76 | *–*12.87 |
|  | ARPGxx1233 | 2006 | dentine | *+*17.04 | *–*12.09 |
| Canadian Arctic (Western Hudson Bay population) | ARARxx1179 | 2008 | dentine | *+*17.52 | *–*11.60 |
|  | ARARxx1190 | 2008 | dentine | *+*16.73 | *–*11.80 |
|  | ARARxx1067 | 2003 | dentine | *+*15.70 | *–*11.21 |
|  | ARARxx1181 | 2008 | dentine | *+*16.65 | *–*10.52 |
|  | ARARxx1121 | 2005 | dentine | ***+*16.75** | ***–*11.94** |
|  | ARARxx1180 | 2008 | dentine | ***+*17.39** | *–*11.64 |
|  | ARARxx1013 | 1999 | dentine | ***+*16.57** | *–*11.43 |
| Gulf of Mexico | TCWC25577 | 1965 | dentine | 20.34 | -8.74 |
|  | TCWC52861 | 1990 | dentine | 18.60 | **-9.71** |
|  | TCWC52862 | 1990 | dentine | 19.16 | -10.72 |
|  | TCWC52866 | 1990 | dentine | 19.66 | **-10.75** |
|  | TCWC52868 | 1990 | dentine | **18.69** | -9.63 |
|  | LSUMZ17024 | 1972 | dentine | 18.24 | -9.44 |
|  | LSUMZ17034 | 1972 | dentine | 18.19 | -10.36 |
|  | LSUMZ16155 | 1971 | dentine | 17.70 | -10.70 |
