# Supplementary material for: Distributions of Arctic and Northwest Atlantic killer whales inferred from oxygen isotopes
Source: Sci Rep. 2021 Mar 24;11:6739. doi: 10.1038/s41598-021-86272-5 (PMC7990931; doi:10.1038/s41598-021-86272-5)
Supplement: Supplementary file 2 — Supplementary Information 2. [file 41598_2021_86272_MOESM2_ESM.docx]

**Supplementary figures**

**
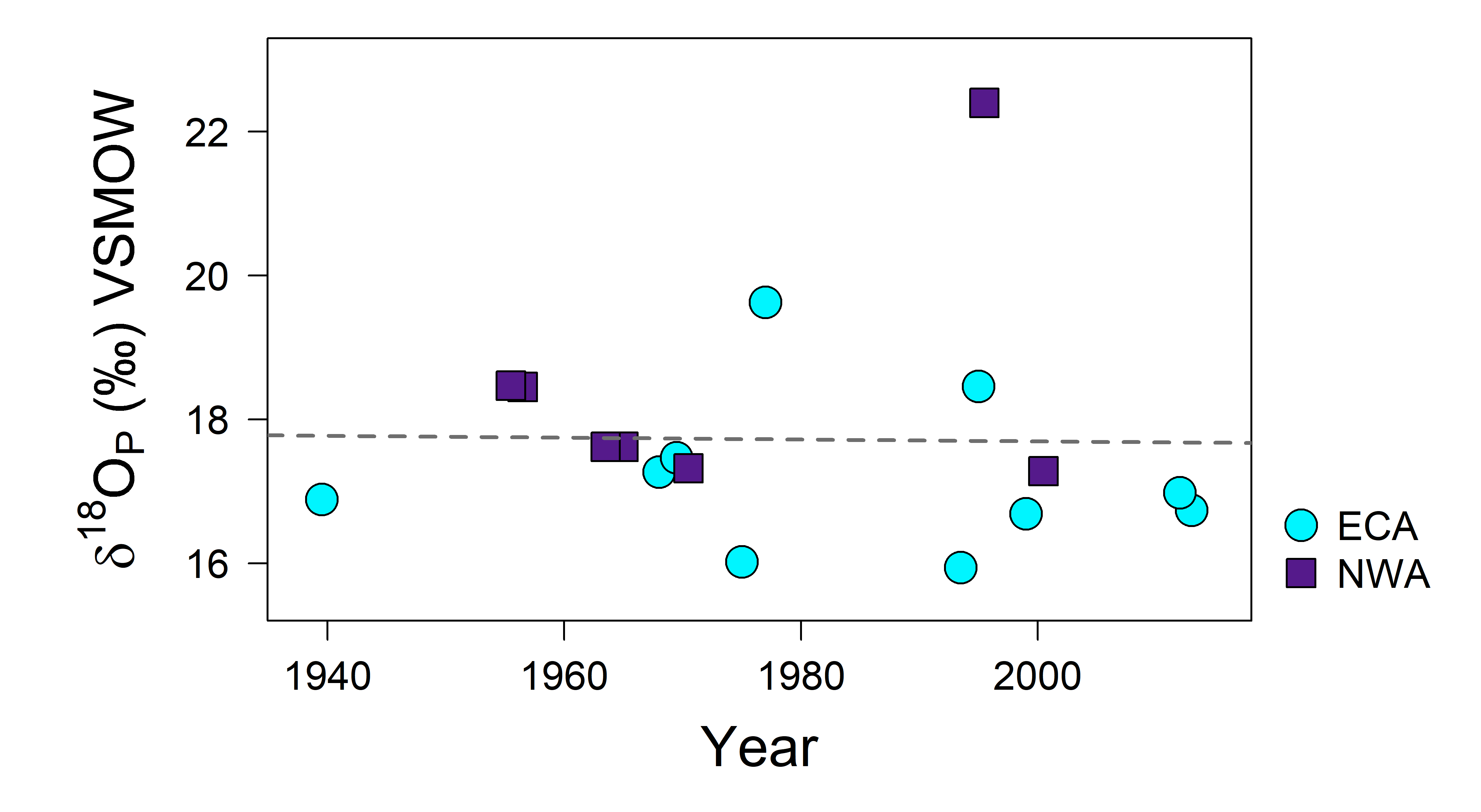
**

Figure S1: Plot of dentine *δ*^18^O_P_ of teeth from eastern Canadian Arctic (ECA; turquoise circles) and Northwest Atlantic (NWA; purple squares) killer whales against calendar year of formation. Since dentine was drilled across all annual growth layer groups (GLGs), calendar year was calculated as the year of death – estimated whale age/2 (see Methods). Linear regression (grey dashed line) showed no significant relationship between *δ*^18^O_P_ and year across the sample period (adjusted R-squared = -0.06633; F_1,15_ = 0.004709; p-value = 0.9462), consistent with other studies showing relatively invariant oceanic *δ*^18^O over even millennial timescales (Clementz et al. 2014).

Clementz, M.T., Fordyce, R.E., Peek, S.L. & Fox, D.L. Ancient marine isoscapes and isotopic evidence of bulk-feeding by Oligocene cetaceans. *Palaeogeogr. Palaeoclimatol. Palaeoecol.* **400**, 28-40 (2014)

**
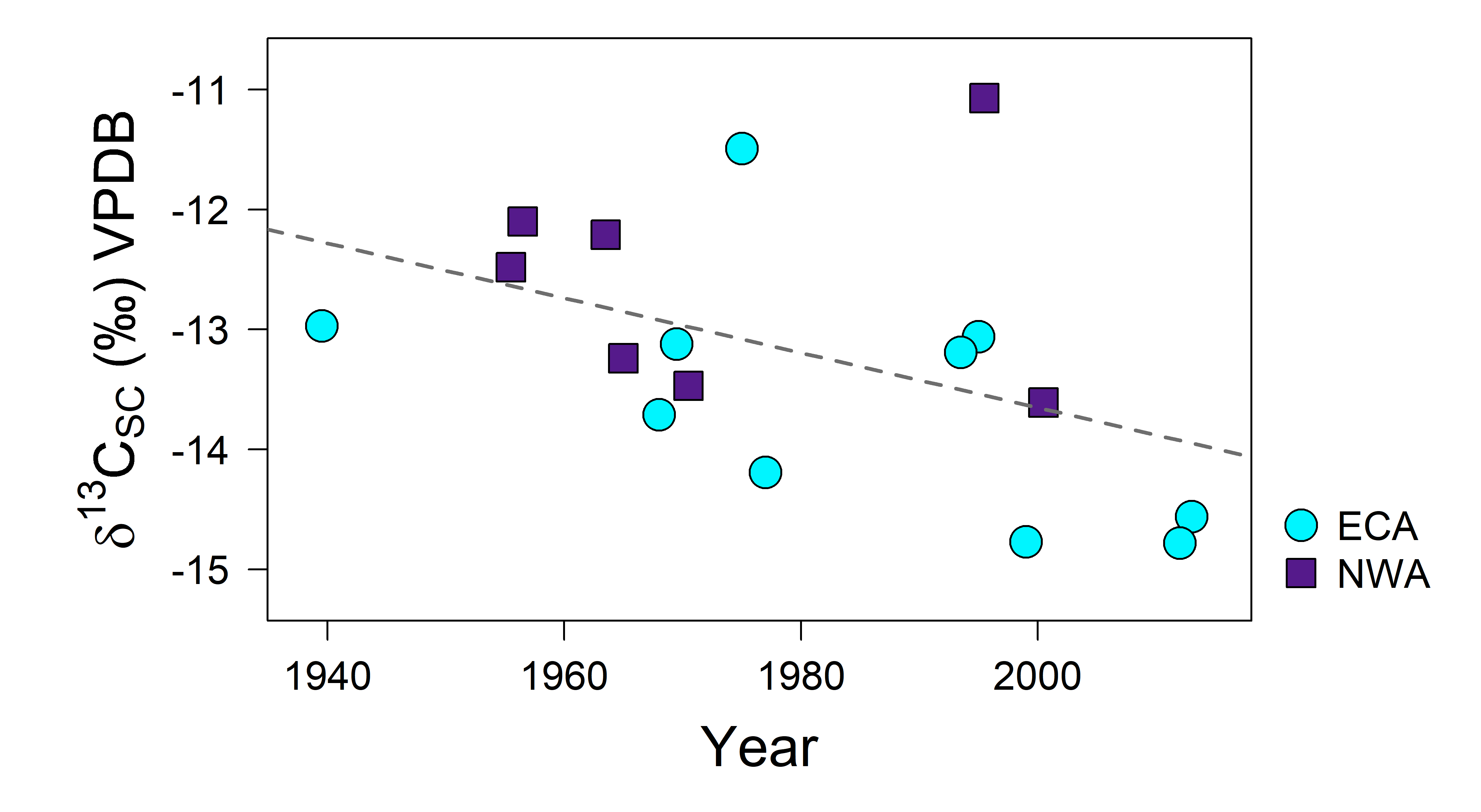
**

Figure S2: Plot of dentine *δ*^13^C_SC_ of teeth from eastern Canadian Arctic (ECA; turquoise circles) and Northwest Atlantic (NWA; purple squares) killer whales against calendar year of formation. Since dentine was drilled across all annual growth layer groups (GLGs), calendar year was calculated as the year of death – estimated whale age/2 (see Methods). Although there is a visible decline in *δ*^13^C_SC_ with time, linear regression (grey dashed line) showed the relationship between *δ*^13^C_SC_ and year was not significant (adjusted R-squared = 0.149; F_1,15_ = 3.81; p-value = 0.07). However, the estimate of the slope (–0.023 ‰ yr^-1^, standard error 0.012) is consistent with the direction and magnitude of *δ*^13^C declines in the North Atlantic attributed to the Suess effect (–0.018 to –0.026 ‰ yr^-1^)^32,33^.
